# Pathway Analyses Identify Novel Variants in the WNT Signaling Pathway Associated with Tuberculosis in Chinese Population

**DOI:** 10.1038/srep28530

**Published:** 2016-06-23

**Authors:** Xuejiao Hu, Juan Zhou, Xuerong Chen, Yanhong Zhou, Xingbo Song, Bei Cai, Jingya Zhang, Xiaojun Lu, Binwu Ying

**Affiliations:** 1 Department of Laboratory Medicine, West China Hospital, Sichuan University, Chengdu 610041, P. R. China; 2 Division of Pulmonary Disease, West China Hospital, Sichuan University, Chengdu 610041, P. R. China

## Abstract

Tuberculosis remains a global public health problem, and its immunopathogenesis is still poorly understood. In this study, 25 single nucleotide polymorphisms (SNPs) in the WNT pathway were evaluated in relation to tuberculosis risk in a Chinese Han discovery set, and 6 candidate susceptible SNPs were further validated in a Chinese Tibetan cohort. Luciferase reporter assay, RT-qPCR and Western blot were used to assess the functionality of the important WNT polymorphisms. Five polymorphisms were associated with tuberculosis susceptibility after Bonferroni correction: *SFRP1* rs4736958, *CTNNB1* rs9859392, rs9870255 and rs3864004 showed decreased tuberculosis risk; *SFRP1* rs7832767 was related to an increased risk (OR = 1.81, 95% CI = 1.30–2.52, *p* = 0.010). Patients with TT genotype of rs4736958 and rs7832767 correlated with higher CRP concentrations (*p* = 0.003, <0.001, respectively). Functional assays revealed that mutant alleles of rs9859392 (G), rs9870255 (C) and rs3864004 (A) were associated with significantly decreased transcriptional activity, lower *CTNNB1* mRNA expression and p-β-catenin level, which were consistent with their effects of decreasing TB risk. Our results provide evidences that WNT pathway polymorphisms influence tuberculosis susceptibility and host immune response to *Mycobacterium tuberculosis*, suggesting that these variations may serve as novel markers for identifying the risk of developing tuberculosis.

Tuberculosis (TB) remains a leading infectious cause of morbidity and mortality worldwide, with an estimated 9.6 million cases and 1.5 million deaths occurring in 2014^1^. More than one-third of the global population is infected with *Mycobacterium tuberculosis* (MTB), however, only 5–10% of infections will develop into clinical TB^2^, suggesting that genetic factors play an important role in the disease outcome after MTB infection. Previous studies have suggested that host genetic variations may contribute to the heterogeneity in disease susceptibility and clinical responses through modulating the immune reaction against MTB^3^,^4^. Over the years, association studies have evaluated many candidate loci that may be associated with susceptibility to TB, including the genes encoding human leukocyte antigen^5^, cytokines and chemokines^6^, toll-like receptor and others^7^. However, most of the studies only investigated a few polymorphisms and were unable to draw definitive conclusions. Thus, more systematic and comprehensive studies are needed to fully clarify the genetic components affecting TB progression. Recently, pathway-based association studies based on the biological functions of related signaling pathway networks were found to be a powerful approach to identify causal genes underlying complex diseases^8^.

The WNT signaling pathway is known as a key transduction cascade governing ontogeny and homeostatic processes^9^, as well as modulating host immune responses against microbial pathogens^10^. It is activated when Wnt proteins bind to Frizzled receptors and co-receptors through a series of cascade reactions, which causes β-catenin co-activating transcription factors to regulate the downstream gene transcription. Antagonists, including the secreted frizzled-related protein (*SFRP*) family, Wnt inhibitory protein one (*WIF1*) and Dickkopf (*DKK*), inhibit WNT pathway signaling by binding to Wnt or Wnt receptors and negatively regulating target gene expression^9^. Recent evidence has indicated that the WNT pathway has an etiological role in TB disease^11^,^12^,^13^,^14^,^15^. Wu *et al*. found that activation of Wnt/β-catenin signaling induced mycobacteria-infected cell apoptosis and promoted pro-inflammatory TNF-α and IL-6 production^11^. A study by Li Y *et al*. revealed that Wnt/β-catenin acted as a negative feedback loop to suppress inflammation stimulated by Mycobacterium bovis Bacillus Calmette-Guerin (BCG), thus alleviating TLR-induced pro-inflammatory responses in alveolar epithelial cells^12^. Neumann *et al*. reported that Wnt/Frizzled signaling was vital in bridging innate and adaptive immunity in MTB infection^13^,^14^. Schaale K. *et al*.^15^ further found that Wnt6 expressed in granulomatous lesions participated in macrophage differentiation and proliferation by driving macrophage polarization toward an M2-like phenotype. The specific molecular mechanism of the WNT pathway in the progression of MTB has garnered interest recently but remains poorly understood. What we do know is that genetic variations within the WNT pathway have been associated with the development of various diseases, such as cancers^16^ and infectious diseases^17^, suggesting that polymorphisms within the WNT pathway may similarly affect TB susceptibility.

China is experiencing a serious epidemic of TB and has the highest annual number of MDR-TB cases worldwide, ranking second among the 22 high-burden countries^18^. In West China, the annual incidence of pulmonary tuberculosis is significantly higher than that in the rest of China, reaching 695 cases per 100,000 inhabitants^19^. Screening a high-risk population among exposed/infected individuals is of great importance in the TB control program established to reduce the burden of disease. We previously investigated a small amount of SNPs in *CTNNB1* gene and *SFRP1* gene in a small sample of Western Chinese, and we identified that two SNPs (*CTNNB1* rs4135385 and *SFRP1* rs7832767) were associated with TB risk, indicating a potential role of these variants in susceptibility to TB^20^. Hence, it is necessary and rational to screen more TB-associated gene loci based on the WNT signaling pathway. To provide a full understanding of the relationship between polymorphisms within the WNT signaling pathway and TB risk, we conducted a 2-stage case-control study (832 cases and 1084 controls in Chinese Han discovery set; 480 cases and 450 controls in Chinese Tibetan validation set) and functional assays for susceptible polymorphisms at both mRNA and protein level.

## Results

### General characteristics of the study population

Demographic characteristics of cases and controls are presented in Table 1. In general, both groups mainly consisted of middle-aged males, and statistically significant differences were observed for BMI and smoking (*p* < 0.001, 0.023, respectively), and not found in BCG status (*p* = 0.089). TB patients involved 367 pulmonary tuberculosis (PTB, 44.11%), 120 extra-pulmonary TB (EPTB, 14.42%) and 345 PTB combined with EPTB (PTB&EPTB, 41.47%).

### Polymorphisms association with tuberculosis in Chinese Han population

#### Single SNP association

Of the 23 SNPs successfully genotyped, six were found to be significantly associated with TB (Tables 2 and 3). In the *CTNNB1* gene, rs9859392, rs9870255 and rs3864004 were significantly different between patients and controls. Subjects with mutant alleles (G, C and A) and dominant model of the three SNPs were associated with decreased TB susceptibility, and the OR ranged from 0.69 to 0.78 (*p* < 0.05). Of the four SNPs in *SFRP1*, two were found to be significant. A reduced risk of TB was related with the mutant C allele, the additive and dominant pattern of rs4736958 with an estimated OR of 0.78, 0.78 and 0.73 (*p* = 0.033, 0.040 and 0.017, after Bonferroni correction). The minor T allele, additive and recessive model of rs7832767 were more frequent in the patients than in the controls (1.30-, 1.29- and 1.81-fold, respectively). A potential beneficial effect was also identified for the additive and dominant model of rs752107 in *WNT3A* [OR_add_ = 0.85 (0.73–0.99), *p*_add_ = 0.040; OR_dom_ = 0.82 (0.68–0.98), *p*_dom_ = 0.033], however, the potential association disappeared after Bonferroni correction in rs752107. No significant differences among the frequencies of the other 18 SNPs were obtained between the patients and the controls.

We further examined whether the six candidate SNPs were preferentially associated with specific tubercular subtypes, as described by Fernando *et al*.^21^. The results are summarized in Table S6. Rs4736958 seemed to have a stronger magnitude of decreased risk with PTB as compared to all forms of TB, and rs9859392, rs9870255 and rs3864004 showed slightly lower OR values with PTB&EPTB as compared to all forms of TB. Rs7832767 was most powerfully associated with a higher risk of PTB&EPTB (OR = 2.19, 95% CI = 1.47–3.26, *p* = 0.001, after Bonferroni correction) in the recessive model.

#### Linkage analysis and haplotype construction

**Figure 1:**
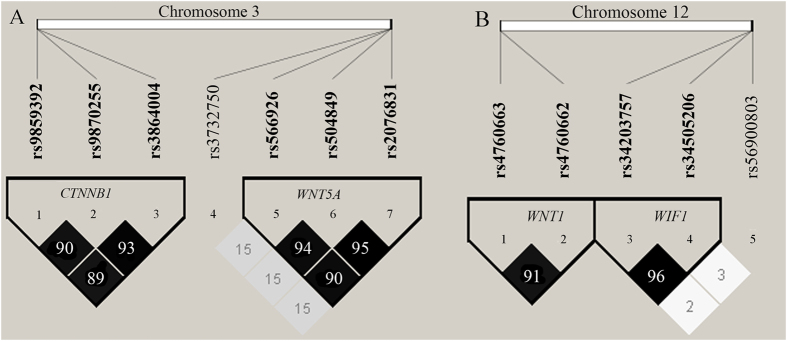
Linkage disequilibrium (LD) plots of SNPs. (**A**) LD results of SNPs in the *CTNNB1* and *WNT5A* genes on chromosome 3. (**B**) LD results of SNPs in the *WNT1* and *WIF1* genes on chromosome 12. Notes: Pairwise *r*^*2*^ values for all pairs of SNPs are presented as percentages in diamonds, and shading from white to black indicates the intensity of *r*^*2*^ from 0 to 1. Strong LD is represented by a high percentage (>80) and a darker square.

Linkage disequilibrium (LD) was estimated by calculating the pairwise *r*^*2*^ coefficient^22^. Figure 1 displays the LD plot of SNPs on the same chromosome. With a pairwise *r*^*2*^ > 0.8, three polymorphisms of *CTNNB1* (rs9859392/rs9870255/rs3864004) and three of *WNT5A* (rs566926/rs504849/rs2076831) on chromosome 3, as well as two SNPs in *WNT1* (rs4760663/rs4760662) and two in *WIF1* (rs34203757/rs34505206) on chromosome 12, were in strong linkage disequilibrium with one another, respectively. We constructed the haplotype by using the SNPs which were in strong LD state from Fig. 1. As seen in Table 4, the [GCA] haplotype frequency of *CTNNB1* was significantly lower in the patients than in the controls for rs9859392/rs9870255/rs3864004 (OR = 0.65, 95% CI = 0.60–0.83, *p* = 0.016, after Bonferroni correction). The distribution of haplotype frequencies of *WNT5A*, *WNT1* and *WIF1* did not differ significantly between the patients and the controls.

#### Gene-gene interaction evaluation

The one-dimensional interactions (including multiplicative and additive interactions) between the six significant SNPs were tested, but they did not yield additional positive information (*p* all > 0.05). High-dimensional interactions among all SNPs were also performed, but no significant interactions were found from the one to four-way models (*p* all > 0.05). Together, gene-gene interactions failed to reveal potential synergistic or antagonistic effects among the polymorphisms in different genes. Details are shown in Tables S7–S9.

#### Correlation between genotypes and clinical phenotypes

**Figure 2:**
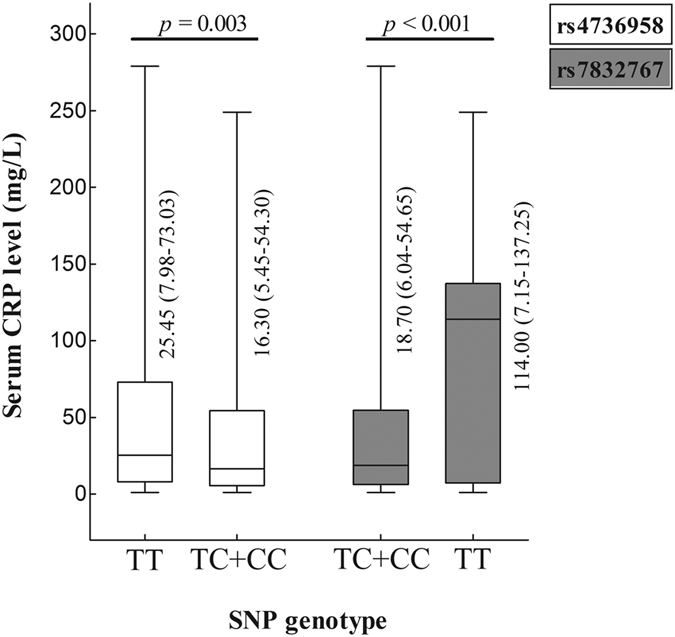
Serum concentration of CRP in relation to polymorphisms of *SFRP1* in TB patients. Notes: rs4736958 (T > C) and rs7832767 (C > T) genotype was stratified based on the significant dominant and recessive model, respectively; rs4736958: TC + CC *vs* TT, rs7832767: TT *vs* TC + CC.

We investigated whether the six candidate susceptibility SNPs affected patients’ clinical manifestations. Common indices of disease severity for TB consisted of symptom intensity (fever, hemoptysis), cavities in the chest radiograph, smear acid-fast and culture of MTB, hemoglobin concentration, lymphocyte count, erythrocyte sedimentation rate, and C-reactive protein (CRP) level, which was regarded as a regular inflammatory marker for host defense and innate immunity^23^. Due to the low frequencies of some minor genotypes, six SNPs were stratified based on the dominant or recessive model. Subgroup analysis attested that rs4736958 and rs7832767 were closely associated with plasma levels of CRP (*p* = 0.003, <0.001), and patients carrying the TT genotype corresponded to higher levels of CRP (Fig. 2). Beyond that, no nominally significant relation between other clinical indices and genotypes were obtained (*p* all > 0.05, data not shown, available on request).

### Candidate SNPs validation in Chinese Tibetan group

Next, we genotyped 6 SNPs (rs4736958, rs7832767, rs9859392, rs9870255, rs3864004 and rs752107) in an independent Chinese Tibetan cohort (480 TB cases and 450 healthy controls). After multiple testing adjustment, only *CTNNB1* SNPs (rs9859392, rs9870255 and rs3864004) were significant referring to frequency distribution, the additive and dominant model (Table 5), which further demonstrated that carriers with mutant genotypes of these SNPs were less susceptible to TB disease.

### Functional exploration of *CTNNB1* polymorphisms

#### Investigation for the effect of polymorphisms on mRNA expression

**Figure 3:**
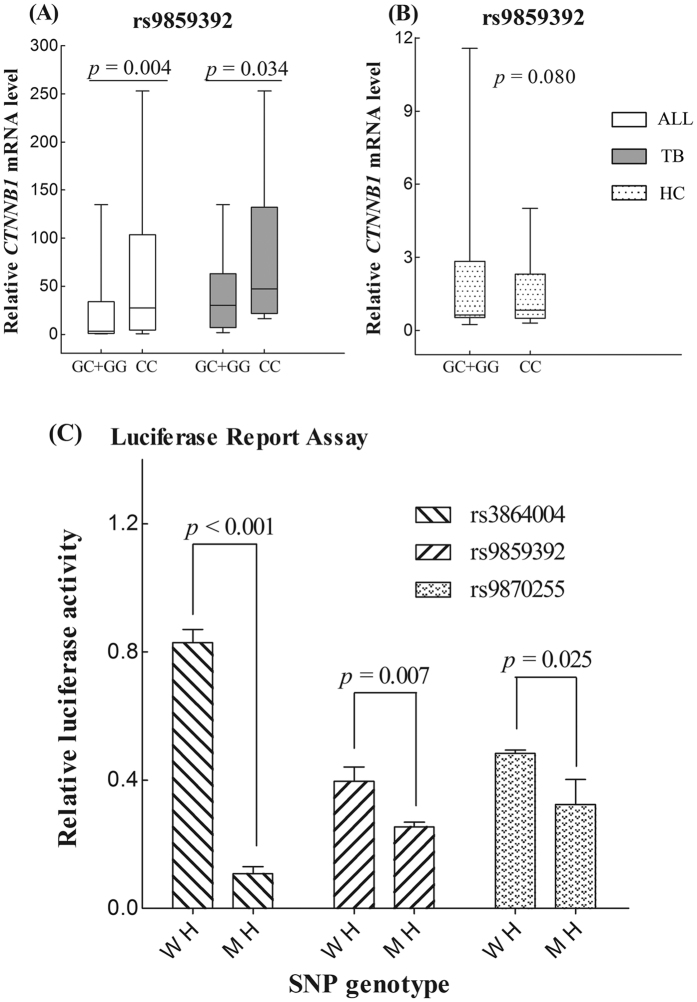
*CTNNB1* polymorphisms affected *CTNNB1* mRNA level and its transcriptional activity. Notes: ALL: all subjects, TB: tuberculosis patients, HC: healthy controls. W H: wild homozygote, M H: mutant homozygote. (**A**,**B**) refer to the *CTNNB1* mRNA levels in relation to different genotypes in all 79 subjects [GC + GG *vs* CC: 3.15 (0.64–33.68) *vs* 27.28 (4.37–103.38)], 50 TB patients [30.24 (6.93–62.69) *vs* 47.08 (21.59–131.98)] and 29 healthy controls [0.64 (0.53–2.85) *vs* 0.83 (0.50–2.31)], respectively. rs3864004, rs9859392 and rs9870255 were in strong linkage disequilibrium, and their genotypes had the same *CTNNB1* mRNA expression level, therefore, we just listed rs9859392 as an example. Subjects were grouped for rs9859392 in *CTNNB1* as genotype with (GG + GC) or without (CC) mutant G allele. (**C**) indicates the effect of three *CTNNB1* polymorphisms on *CTNNB1* promoter transcriptional activity in 293T cells. Each bar represents the mean of triplicate transfected plates plus standard deviation (rs3864004: 0.83 ± 0.04 *vs* 0.09 ± 0.01; rs9859392: 0.40 ± 0.05 *vs* 0.25 ± 0.02; rs9870255: 0.48 ± 0.01 *vs* 0.30 ± 0.08).

We further explored the effect of six TB-associated SNPs on gene expression. The mRNA levels of candidate susceptibility genes (*CTNNB1*, *SFRP1* and *WNT3A*) from a subset of 50 patients and 29 controls were compared, not only between the cases and the controls but also in different genotypes. The mRNA expressions of all three genes were significantly higher in the patients than in the controls [*CTNNB1*: 40.16 (20.13–107.17) *vs* 0.77 (0.52–2.53), *p* < 0.001; *SFRP1*: 10.22 (1.58–26.36) *vs* 2.23 (0.31–7.68), *p* = 0.008; *WNT3A:* 11.16 (2.24–58.23) *vs* 0.84 (0.39–2.37)*, p* < 0.001], implying that their roles in the regulation of TB progression. The dominant pattern of three significant SNPs in *CTNNB1* (rs3864004, rs9859392 and rs9870255) was associated with obviously decreased *CTNNB1* mRNA levels both in all 79 subjects and in 50 TB patients (*p*_*all*_ = 0.004, *p*_*TB*_ = 0.034), however, no association was found in healthy controls (*p*_*HC*_ = 0.080). Figure 3(A,B) show the example of the comparison of *CTNNB1* mRNA expression based on the dominant genotype model of rs9859392 in these three groups. In addition, similarly significant effects were not observed in the three significant SNPs in *SFRP1* and *WNT3A* (*p* all > 0.05, data not shown, available on request).

#### Influence of polymorphisms on transcriptional activity of CTNNB1 gene

To assess the effects of SNPs rs3864004, rs9859392 and rs9870255 on *CTNNB1* gene transcription, we analyzed the transcriptional activity of its promoter and compared the activities of mutant and wild allele using luciferase report assay. As shown in Fig. 3(C), the pGL3-mutant homozygote reporters have significantly decreased luciferase activity as compared to pGL3-wild homozygote (9.22-folds for rs3864004, *p* < 0.001; 1.60-folds for rs9859392 and rs9870255, *p* = 0.007, 0.025, respectively). This finding indicates that these three SNPs are functional, and the substitution of the mutant allele for the wild allele can suppress *CTNNB1* promoter transcriptional activity.

#### Effect of CTNNB1 polymorphisms on its protein expression

**Figure 4:**
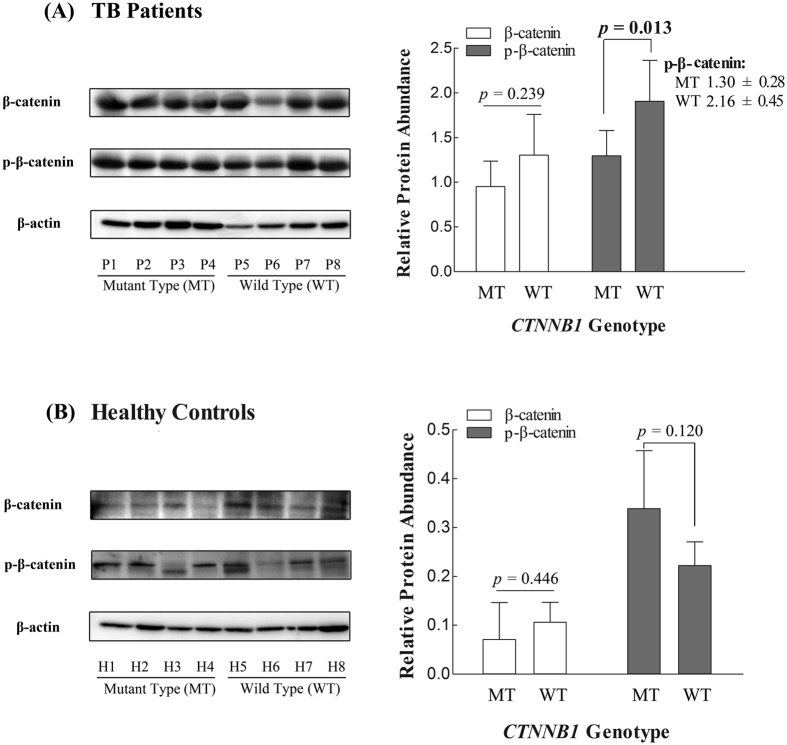
Effect of *CTNNB1* polymorphisms on its endogenetic protein expression. Notes: P1-P4: TB patients with mutant genotype of *CTNNB1* polymorphisms (rs9859392/rs9870255/rs3864004), i.e. subjects with haplotype [GCA]. P5-P8: TB patients with wild genotype of *CTNNB1* polymorphisms, i.e. subjects with haplotype [CGG]. Similarly, H1-H4 and H5-H6 refer to healthy controls with mutant genotype and wild genotype of *CTNNB1* polymorphisms, respectively. (**A**,**B**) protein levels of β-catenin, phosphorylated β-catenin (p-β-catenin) and β-actin were determined by Western blot in the PBMC of 8 TB patients and 8 healthy controls, respectively. Each bar represents the mean grey value of each group plus standard deviation.

To verify whether the three SNPs could influence the endogenetic expression of *CTNNB1* gene, we examined the protein products (β-catenin and p-β-catenin) in peripheral blood mononuclear cells (PBMC) of 16 TB patients and healthy controls. As shown in Fig. 4, compared with healthy controls, tuberculosis patients had significantly increased β-catenin and p-β-catenin protein expression (darker bands). In order to adjust the discordance of cell quantities among samples, relative gray intensity analysis of β-catenin and p-β-catenin protein bands was normalized by its β-actin expression and was further calculated using Image J software (NIH, MD, USA). Levels of p-β-catenin protein were significantly decreased in patients with mutant genotype (haplotype [GCA] for rs9859392/rs9870255/rs3864004) as compared with patients carrying wild genotype (haplotype [CGG]), indicating the similar trend with lower mRNA expression level and reduced TB risk.

## Discussion

The present study identified five SNPs within the WNT pathway (*SFRP1* rs4736958 and rs7832767; *CTNNB1* rs9859392, rs9870255 and rs3864004) that were associated with TB susceptibility. Further explorations preliminarily unveiled a relationship between two *SFRP1* SNPs and serum CRP levels and a modulatory effect of three *CTNNB1* polymorphisms on its mRNA and protein expression. To our knowledge, this is the first study to comprehensively interrogate the relationship between WNT pathway-based SNPs and TB risk, providing insight into the detailed mechanism for the pathogenesis of TB and a clue for clinical biomarker screening.

The *CTNNB1* gene encodes the β-catenin protein, dually functioning in the coordination of cell adhesion and gene transcription. Compelling studies have indicated that aberrant β-catenin participates in an abnormal immune response to pathogens^10^ and disease course, including tuberculosis^13^. Recently, researchers have linked *CTNNB1* mutations to the susceptibility and prognosis of various cancers^24^. However, the impact of the *CTNNB1* variations on TB has not yet been corroborated. In four *CTNNB1* SNPs, we identified mutant alleles of rs9859392 (G), rs9870255 (C) and rs3864004 (A) that might be protective factors for TB according to our discovery and validation cohorts. Haplotype analysis revealed a powerful reduced-risk haplotype, [GCA], composed of these minor alleles, thus supporting the reliability of single locus results and providing evidence of a synergistic beneficial effect of these SNPs on TB infection. Higher *CTNNB1* expressions were observed in TB patients, pointing to a critical role of *CTNNB1* in host immune response to MTB. Subsequent functional investigations found that mutant alleles at these SNPs significantly decreased *CTNNB1* promoter transcriptional activity and correlated with lower *CTNNB1* mRNA and phosphorylated β-catenin protein expression levels, identical to their effects of decreasing TB risk. Similar correlations were also reported by Sungmin Bae *et al*.^25^, who demonstrated that β-catenin haplotypes formed by these polymorphisms affected mRNA expression and were associated with asthma risk. Taken together, these observations supported the idea that these SNPs resulted in different recruits of transcription factors to *CTNNB1* gene promoter region, which modulated *CTNNB1* mRNA and protein expression and leaded to abnormal activation of the WNT pathway, participating in the pathogenesis of disease. Further functional experiments are warranted to confirm and elaborate these findings.

*SFRP1* belongs to a well-established antagonist of the WNT pathway, and molecular abnormalities in its expression have been amply demonstrated to be involved in the development of carcinoma^26^. As a novel factor modulating inflammation^27^, *SFRP1* may play an important role in the pathogenesis of TB. We explored five *SFRP1* polymorphisms and found two (rs4736958 and rs7832767) that were significantly association with TB risk in Chinese Han population, but not in Tibetan group. This suggests that ethnic heterogeneity of these polymorphisms might exist among different populations, which is worthy of further investigation in other populations. Notably, we found that the risk genotype TT in these two SNPs was related to higher CRP levels, indicating that patients carrying the risk genotype TT may have a higher inflammatory state. When the two SNPs were analyzed for a possible association with gene expression, no significant differences were found, suggesting that these polymorphisms may not influence *SFRP1* mRNA expression. Rs7832767 is located in the intron region, and rs4736958 lies in the 3′ untranslated region of *SFRP1*, neither of which directly code for the Sfrp1 protein, even though there seem to be some indirect links. As a tagSNP, rs7832767 may be in linkage disequilibrium with a functional mutation that controls the expression or function of *SFRP1*. According to the bioinformatics tool SNPinfo (NIH, SNPinfo Web Server. Available at: http://snpinfo.niehs.nih.gov/), rs4736958 is located in the seed binding site of miR-9 and *SFRP1*. The high-affinity miR-9-binding motif is predicted to be abrogated by an rs4736958 C-to-T transition, ultimately altering the regulation of miR-9 to inflammatory responses against invasive pathogens, as evidenced by Thulin *et al*.^28^. Although we do not currently know if these speculations conform to concrete mechanisms, ongoing studies will address these questions.

In this research, we found that rs752107 was associated with a reduced risk for TB in allele distribution, the additive and dominant model. This association did not remain after Bonferroni correction or in the validation cohort, therefore, we determined that rs752107 was not TB risk-related locus, even though other independent studies are needed. With regard to the rest of the SNPs and genes analyzed, no substantial associations with TB risk were obtained, suggesting that these polymorphisms may not predispose one to the disease. However, we cannot completely exclude their possible role as putative TB-risk genes on account of the imperceptible scrutiny of genotype frequency or mild effect of these polymorphisms. Despite these irrelevant results, we expect that our work will help to elucidate the genetic variations involved in the pathogenesis of TB.

Sample size in the present study was still limited, especially for some stratifications, therefore, some polymorphisms with mildly significant effects might not be detected. On the other hand, our study lacks some demographic information on the Chinese Tibetan cohort, and our results should be validated in larger population-based studies with more detailed information.

In conclusion, this study investigated the pathogenesis of TB from the perspective of pathway-based polymorphisms. Five SNPs in the WNT pathway (*SFRP1* rs4736958 and rs7832767; *CTNNB1* rs9859392, rs9870255 and rs3864004) were associated with TB susceptibility. Two SNPs in *SFRP1* were correlated with serum CRP levels, and three *CTNNB1* polymorphisms had a striking effect on *CTNNB1* promoter transcriptional activity and modulated *CTNNB1* mRNA and protein expression. Our findings suggest that genetic variations within the WNT pathway may play a significant role in TB risk and host inflammatory status.

## Methods

### Study population and samples

Discovery set: a total of 1916 participants (832 active TB cases and 1084 gender- and age-matched controls) were enrolled in this study. Cases were newly diagnosed TB patients consecutively recruited from the West China Hospital of Sichuan University between November 2011 and October 2015. Eligible cases were clinically diagnosed by experienced specialists using microbiological or pathological evidence of TB (smear/culture/TB-DNA positive) to confirm MTB infection. Patients who were HIV-positive, had other lung or infectious diseases or had an immunosuppressive condition were excluded. Controls were recruited from healthy blood donors with a negative interferon gamma release assay (TB-IGRA) result, no history of TB and a normal physical examination. All subjects in this study were unrelated Han Chinese in West China. Demographic and clinical data of cases and controls were obtained from interviews or extracted from medical records. Validation set was consist of 480 Tibetan cases and 450 gender- and age-matched Tibetan controls. This study was approved the Clinical Trials and Biomedical Ethics Committee of West China Hospital, Sichuan University [Reference No. 198 (2014)], and the methods were carried out in accordance with the approved guidelines. Informed consent was obtained from all individuals.

### Candidate loci selection and genotyping

Key genes in the WNT signaling pathway were selected based on their biological functions as core components of this pathway, including *CTNNB1*, *WIF1*, *DKK1*, *SFRP1*, *WNT1*, *WNT3A* and *WNT5A*^9^,^10^,^11^,^12^,^13^,^14^,^15^. We selected candidate SNPs for this study through a thorough search of the International HapMap project, dbSNP database, 1000 Genomes database. SNPs were included if they were located in potential functional regions (exon, promoter and untranslated region) and could well represent those with minor allele frequency (MAF) >0.05 in a Chinese Han Beijing population. Finally, a total of 25 SNPs were selected for subsequent genotyping (Table S1).

Genomic DNA was extracted from 2- to 3-ml peripheral blood using a QIAamp^®^ DNA blood Mini kit (Qiagen, Hilden, Germany). SNPs were genotyped using the MassARRAY platform (Sequenom, CA, USA) with primers and probes (Table S2) as described by Buetow *et al*.^29^, and SNP genotyping in validation cohort was performed by Shanghai Genesky Bio-Tech Genetic Core Lab (Shanghai, China) using multiplex ligation detection reaction as described by Thomas *et al*.^30^ (Primers and probes information presented in Tables S3 and S4). The laboratory staff was blinded to the case-control status of the samples. We included water (HPLC grade) as the negative control in each of the 96-well or 384-well plates. Fifty random samples were genotyped in duplicate with a concordance rate of 100%. SNPs with a clear genotype clustering and a call rate >95% were considered for further analysis.

### Measurement of mRNA expression

We collected an additional 3 ml of peripheral blood from 85 participants and isolated peripheral blood mononuclear cells (PBMC) from 79 of them for mRNA measurement. Total RNA was isolated using TRIzol reagent (Invitrogen, CA, USA) and then converted to complementary DNA (cDNA) using Omniscript^®^ Reverse Transcription Kit (Qiagen, CA, USA). The final 10-μl-volume reaction for RT-qPCR included 5 μl of SYBR^®^ Premix Ex Taq™ II (Takara, Dalian, China), 0.8 μl of 10 μM forward and reverse primer (Table S5), 3.2 μl of water and 1 μl of cDNA template. RT-qPCR amplification was carried out in a LightCycler^®^ 480 Real-Time PCR System (Roche Diagnostics, NJ, USA). The reaction setting consisted of an initial denaturation of 10 min at 95 °C and amplification for 40 cycles by denaturing at 95 °C for 20 s, annealing at 56–62 °C for 30 s and extension at 72 °C for 25 s. The samples were denatured at 95 °C for 30 s and then heated to 65 °C for 30 s at a rate of 0.2 °C/s. Fluorescence was measured to generate a denaturing curve of the amplified products. Raw data were analyzed using Gene Scanning v1.2 software. For each sample, the mRNA expression was normalized to the endogenous control *GAPDH* and calculated according to the 2^−ΔΔCT^ method.

### Luciferase report assay

Since SNP rs9859392, rs3864004, rs9870255 were suggested to be the most effective variant within or near the promoter of *CTNNB1*, we further tested their effects on the promoter activity of the gene using a Dual-Luciferase Reporter Assay System (Promega, WI, USA). Briefly, PCR fragments containing either wide or mutant allele of these SNPs were amplified using the following primers (the sequence in bold is a KpnI or XhoI restriction site): rs9870255: Sense: 5′-GG**GGTACC**CAGTGCTAGGGTGGTGAGTG-3′, Antisense: 5′-CCG**CTCGAG**AATCCCATCATGCAGGGTCC-3′; rs9859392: Sense: 5′-GG**GGTACC**ATGGACAATCCCTTGAAGCAAC-3′, Antisense: 5′-CCG**CTCGAG**TCCTTGATAAAAATGAGGAAAGTGG-3′; rs3864004: Sense: 5′-CGG**GGTACC**GCTCTGGAGCTAATCCATTTCCA-3′, Antisense: 5′-CCG**CTCGAG**CTTATAAGTCGCGCAGAAGCC-3′. The cycling program consisted of 98 °C for 2 min, followed by 35 cycles of 98 °C for 10 s, 55 °C for 15 s and 72 °C for 60 s. PCR products were then cloned into the pGL3-Basic vector or pGL3-Promoter vector. All reporter constructs were verified by direct sequencing. For luciferase assays, 293T cells were plated in a 24-well plate and allowed to grow for 1 day prior to transfection (60–70% confluence). 293T cells were cultured in Dulbecco’s modified Eagle’s medium (DMEM, Gibco, USA) containing 10% heat-inactivated fetal bovine serum (Biowest, France). Transfection was performed using Lipofectamine 2000 Reagent (Invitrogen), with 0.2 μg of a given pGL-3 construct and 20 ng of renilla vector (pRLTK, as internal control) per well were co-transfected. After 48 h, cells were lysed in 16× Passive Lysis Buffer, and 30 μl of lysate was used to detect firefly luciferase (LAR II) and renilla luciferase (stop and glow buffer) activity. Fluorescence intensity was measured using BioTek Synergy 4 multi-mode microplate reader (BioTek Instruments Inc., USA). The relative luciferase activity was normalized to the cotransfected control Renilla luciferase. Each construct was transfected in triplicates and assayed in duplicate.

### Western blot

PBMC from TB patients and healthy controls were freshly isolated using Human Lymphocyte Separation Tube Kit (Dakewe Biotech company limited, Shenzhen, China), and then proteins were extracted proteins were extracted in RIPA buffer and quantified by the BCA protein assay kit (Bio-Rad). The resulted proteins were subjected to 10% SDS-PAGE and transferred to PVDF membranes (Millipore). The membrane was blocked with 5% bovine serum albumin (BSA)-PBS and was incubated with rabbit anti-β-catenin and p-β-catenin (diluted 1:1000, Cell Signaling Technology, MA, USA) at 4 °C overnight, respectively. The blots were incubated with secondary antibody (diluted 1:2000; Zhongshan Biological) conjugated to horseradish peroxidase for 2 h. β-Actin was used as an internal control. Blots were visualized by enhanced chemiluminescence reagents (Amersham Biosciences, Shanghai, China) and analyzed with Image J Software (NIH, MD, USA).

### Statistical analysis

Univariate analysis was used to analyze categorical variables with a Chi-square test and continuous variables with the Mann-Whitney U test. Hardy-Weinberg equilibrium (HWE) among the controls was evaluated using Fisher’s exact test. SNPs with monomorphism and significant HWE deviation were excluded from analysis. Associations between SNPs and TB risk were evaluated on the basis of allele frequency distributions and genetic models (additive, dominant and recessive model). Wild or major allele of each SNP was set as the reference, and odds ratio (OR) and 95% confidence interval (CI) were derived from unconditional logistic regression models, adjusting for age, gender and BMI. Linkage disequilibrium was assessed using Haploview v4.2^31^. Haplotype frequencies and associations were calculated based on the expectation-maximization clustering algorithm using this software. The one-dimensional interactions between two associated SNPs were evaluated by logistic regression for multiplication interactions using the method suggested by Andersson *et al*.^32^. High-dimensional interactions among multiple SNPs were assessed using general multifactor dimensionality reduction (GMDR) software^33^.

Analyses were performed using SPSS v19.0, PLINK v1.07, Haploview v4.2 and GMDR. All tests were two-sided, and nominal statistical significance was established at an alpha level of 0.05 unless stated otherwise.

## Additional Information

**How to cite this article**: Hu, X. *et al*. Pathway Analyses Identify Novel Variants in the WNT Signaling Pathway Associated with Tuberculosis in Chinese Population. *Sci. Rep.* **6**, 28530; doi: 10.1038/srep28530 (2016).

## Supplementary Material

Supplementary Information

## Figures and Tables

**Table 1:** Demographic characteristics of study participants in Chinese Han population.

Characteristics	TB^a^ (n = 832)	HC^b^ (n = 1084)	*P*
Age, mean ± SD (years)	41.10 ± 19.04	40.54 ± 10.69	0.574
Male/female	496/336	629/455	0.484
BMI (kg/m^2^)	20.99 ± 2.55	23.03 ± 2.06	**<0**.**001**
BCG scar n (%)			0.089
Yes	376 (45.19)	453 (41.79)	
No	361 (43.39)	524 (48.34)	
Uncertain	95 (11.42)	107 (9.87)	
Smoking n (%)			**0**.**023**
Smoking	262 (31.49)	283 (26.10)	
Ever Smoking	135 (16.23)	207 (19.09)	
Nonsmoking	435 (52.28)	594 (54.81)	
TB subtype n (%)			
PTB^c^	367 (44.11)		
EPTB^d^	120 (14.42)		
PTB & EPTB	345 (41.47)		

^a^Tuberculosis patients.

^b^Healthy controls.

^c^Pulmonary tuberculosis (PTB).

^d^Extra pulmonary tuberculosis (EPTB).

**Table 2:** Comparison of frequency distributions of WNT pathway polymorphisms in Chinese Han population.

Gene	SNP	Group	Allele n (%)		OR (95% CI)	P*	P**	Genotype n (%)			P*	P**
			1	2				11	12	22		
*CTNNB1*	**rs9859392**	TB	316 (19.41)	1312 (80.59)	**0**.**75** (**0**.**64–0**.**88**)	**4**.**07 ×10**^**−4**^	**0**.**009**	38 (4.67)	240 (29.48)	536 (65.85)	**6**.**57 × 10**^**−4**^	**0**.**013**
	C > G	HC	515 (24.25)	1609 (75.75)				60 (5.65)	395 (37.19)	607 (57.16)		
	**rs9870255**	TB	323 (19.84)	1305 (80.16)	**0**.**76** (**0**.**65–0**.**89**)	**7**.**66 × 10**^**−4**^	**0**.**018**	34 (4.18)	255 (31.32)	525 (64.50)	**0**.**003**	0.092
	G > C	HC	522 (24.46)	1612 (75.54)				64 (6.00)	394 (36.92)	609 (57.08)		
	**rs3864004**	TB	322 (19.80)	1304 (80.20)	**0**.**77** (**0**.**66–0**.**91**)	**0**.**001**	**0**.**033**	36 (4.43)	250 (30.75)	527 (64.82)	**0**.**007**	0.149
	G > A	HC	509 (24.19)	1595 (75.81)				67 (6.37)	375 (35.65)	610 (57.98)		
*WIF1*	rs34203757	TB	280 (17.11)	1356 (82.89)	0.92 (0.78–1.09)	0.327		28 (3.47)	224 (27.72)	556 (68.81)	0.592	
	C > G	HC	385 (18.14)	1737 (81.86)				44 (4.19)	297 (28.31)	708 (67.49)		
	rs34505206	TB	270 (16.52)	1364 (83.48)	0.87 (0.74–1.03)	0.116		29 (3.55)	212 (25.95)	576 (70.50)	0.311	
	C > G	HC	390 (18.50)	1718 (81.50)				46 (4.36)	298 (28.27)	710 (67.37)		
	rs56900803	TB	200 (12.27)	1430 (87.73)	1.00 (0.82–1.22)	0.987		15 (1.84)	170 (20.86)	630 (77.30)	0.359	
	G > T	HC	260 (12.25)	1862 (87.75)				12 (1.13)	236 (22.24)	813 (76.63)		
*DKK1*	rs11001553	TB	119 (7.48)	1471 (92.52)	1.04 (0.81–1.34)	0.737		7 (0.88)	105 (13.21)	683 (85.91)	0.925	
	C > T	HC	156 (7.2)	2012 (92.80)				8 (0.73)	140 (12.92)	936 (86.35)		
	rs1896367	TB	494 (30.31)	1136 (69.69)	0.98 (0.85–1.13)	0.792		72 (8.83)	350 (42.94)	393 (48.23)	0.272	
	G > A	HC	662 (30.71)	1494 (69.29)				115 (10.67)	432 (40.07)	531 (49.26)		
	rs1896368	TB	623 (38.46)	997 (61.54)	0.96 (0.84–1.09)	0.499		114 (14.07)	395 (48.77)	301 (37.16)	0.131	
	G > A	HC	825 (39.55)	1261 (60.45)				178 (17.06)	469 (44.97)	396 (37.97)		
*SFRP1*	rs3242	TB	101 (6.17)	1535 (93.83)	0.96 (0.74–1.25)	0.776		13 (1.59)	75 (9.17)	730 (89.24)	0.788	
	C > T	HC	138 (6.4)	2018 (93.60)				7 (0.65)	124 (11.50)	947 (87.85)		
	**rs4736958**	TB	471 (28.79)	1165 (71.21)	**0**.**78** (**0**.**69–0**.**92**)	**0**.**001**	**0**.**033**	79 (9.66)	313 (38.26)	426 (52.08)	**0**.**003**	0.092
	T > C	HC	727 (33.66)	1433 (66.34)				125 (11.57)	477 (44.17)	478 (44.26)		
	rs72643819	TB	648 (39.80)	980 (60.20)	1.12 (0.98–1.28)	0.088		154 (18.92)	340 (41.77)	320 (39.31)	0.063	
	G > T	HC	798 (37.08)	1354 (62.92)				160 (14.87)	478 (44.42)	438 (40.71)		
	**rs7832767**	TB	483 (29.45)	1157 (70.55)	**1**.**30** (**1**.**11–1**.**48**)	**6**.**93 × 10**^**−4**^	**0**.**016**	89 (10.85)	305 (37.20)	426 (51.95)	**8**.**81 × 10**^**−4**^	**0**.**021**
	C > T	HC	529 (24.54)	1627 (75.46)				68 (6.31)	393 (36.46)	617 (57.24)		
*WNT1*	rs4760662	TB	808 (49.88)	812 (50.12)	1.01 (0.88–1.15)	0.924		210 (25.93)	388 (47.90)	212 (26.17)	0.778	
	C > T	HC	1064 (49.72)	1076 (50.28)				267 (24.95)	530 (49.53)	273 (25.52)		
	rs4760663	TB	778 (47.67)	854 (52.33)	1.01 (0.89–1.14)	0.922		204 (24.85)	380 (46.28)	237 (28.87)	0.297	
	C > T	HC	1036 (47.83)	1130 (52.17)				248 (22.90)	540 (49.86)	295 (27.24)		
*WNT3A*	rs13373831	TB	339 (20.60)	1307 (79.40)	0.94 (0.80–1.10)	0.409		43 (5.22)	253 (30.74)	527 (64.04)	0.423	
	A > G	HC	464 (21.70)	1674 (78.30)				42 (3.93)	380 (35.55)	647 (60.52)		
	rs708113	TB	590 (36.88)	1010 (63.12)	1.04 (0.91–1.19)	0.607		118 (14.75)	354 (44.25)	328 (41.00)	0.288	
	T > A	HC	760 (36.05)	1348 (63.95)				132 (12.52)	496 (47.06)	426 (40.42)		
	rs74672629	TB	164 (9.98)	1480 (90.02)	0.92 (0.74–1.14)	0.440		7 (0.85)	150 (18.25)	665 (80.90)	0.530	
	C > G	HC	226 (10.75)	1876 (89.25)				7 (0.67)	212 (20.17)	832 (79.16)		
	**rs752107**	TB	398 (24.54)	1224 (75.46)	**0**.**86** (**0**.**74**–**1**.**00**)	**0**.**047**	> 0.999	45 (5.55)	308 (37.98)	458 (56.47)	0.101	
	C > T	HC	590 (27.44)	1560 (72.56)				69 (6.42)	452 (42.05)	554 (51.53)		
*WNT5A*	rs2076831	TB	597 (36.67)	1031 (63.33)	1.04 (0.91–1.19)	0.605		112 (13.76)	373 (45.82)	329 (40.42)	0.727	
	C > G	HC	768 (35.85)	1374 (64.15)				148 (13.82)	472 (44.07)	451 (42.11)		
	rs3732750	TB	189 (11.60)	1441 (88.40)	1.23 (1.00–1.51)	0.055		9 (1.10)	171 (20.98)	635 (77.91)	0.143	
	G > A	HC	208 (9.67)	1944 (90.33)				7 (0.65)	194 (18.03)	875 (81.32)		
	rs504849	TB	603 (36.90)	1031 (63.10)	1.00 (0.87–1.14)	0.971		144 (17.00)	375 (44.27)	328 (38.73)	0.999	
	A > G	HC	788 (36.96)	1344 (63.04)				149 (13.98)	490 (45.97)	427 (40.06)		
	rs566926	TB	612 (38.25)	988 (61.75)	1.01 (0.89–1.16)	0.849		114 (14.25)	384 (48.00)	302 (37.75)	0.268	
	A > C	HC	812 (37.94)	1328 (62.06)				169 (15.79)	474 (44.30)	427 (39.91)		

SNPs were bolded and underlined if they showed a significance of *p* < 0.05; “1” designates the mutant allele and “2” designates the wild allele; 11 = mutant homozygote, 12 = heterozygote, 22 = wild homozygote; OR = odds ratio, followed by 95% CI in parentheses. P*: *p* value after adjusting for age, gender and BMI; P**: *p* value after Bonferroni correction.

**Table 3:** Association of WNT pathway polymorphisms with TB risk in Chinese Han population.

Gene	SNP	Additive Model			Dominant Model			Recessive Model		
		OR	P*	P**	OR	P*	P**	OR	P*	P**
***CTNNB1***	**rs9859392C** > **G**	**0**.**76** (**0**.**65**–**0**.**88**)	**4**.**00 × 10**^**−4**^	**0**.**010**	**0**.**69** (**0**.**57**–**0**.**84**)	**1**.**35 × 10**^**−4**^	**0**.**003**	0.82 (0.54–1.24)	0.344	
	**rs9870255G** > **C**	**0**.**77** (**0**.**65**–**0**.**90**)	**8**.**00 × 10**^**−4**^	**0**.**019**	**0**.**73** (**0**.**61**–**0**.**88**)	**0**.**001**	**0**.**026**	0.68 (0.45–1.05)	0.079	
	**rs3864004G** > **A**	**0**.**78** (**0**.**67**–**0**.**91**)	**0**.**002**	**0**.**039**	**0**.**75** (**0**.**62**–**0**.**90**)	**0**.**003**	0.063	0.68 (0.45–1.03)	0.071	
*WIF1*	rs34203757C > G	0.92 (0.78–1.09)	0.340		0.92 (0.76–1.13)	0.434		0.81 (0.50–1.31)	0.391	
	rs34505206C > G	0.88 (0.74–1.04)	0.130		0.86 (0.71–1.05)	0.146		0.81 (0.50–1.30)	0.374	
	rs56900803G > T	1.00 (0.82–1.22)	0.990		0.96 (0.77–1.20)	0.731		1.64 (0.76–3.52)	0.205	
*DKK1*	rs11001553C > T	1.04 (0.82–1.33)	0.740		1.04 (0.80–1.35)	0.787		1.20 (0.43–3.31)	0.732	
	rs1896367G > A	0.98 (0.86–1.13)	0.790		1.04 (0.87–1.25)	0.655		0.81 (0.60–1.11)	0.186	
	rs1896368G > A	0.96 (0.84–1.09)	0.500		1.04 (0.86–1.25)	0.722		0.80 (0.62–1.03)	0.079	
***SFRP1***	rs3242C > T	0.97 (0.75–1.24)	0.790		0.87 (0.65–1.16)	0.347		2.47 (0.98–6.22)	0.055	
	**rs4736958T** > **C**	**0**.**78** (**0**.**70**–**0**.**92**)	**0**.**002**	**0**.**040**	**0**.**73** (**0**.**61**–**0**.**88**)	**7**.**41 × 10**^**−4**^	**0**.**017**	0.82 (0.61–1.10)	0.183	
	rs72643819G > T	1.11 (0.98–1.26)	0.100		1.06 (0.88–1.28)	0.544		**1**.**34** (**1**.**05**–**1**.**70**)	**0**.**019**	0.447
	**rs7832767C** > **T**	**1**.**29** (**1**.**10**–**1**.**46**)	**0**.**001**	**0**.**023**	**1**.**26** (**1**.**03**–**1**.**49**)	**0**.**022**	0.505	**1**.**81** (**1**.**30**–**2**.**52**)	**4**.**29 × 10**^**−4**^	**0**.**010**
*WNT1*	rs4760662C > T	1.01 (0.89–1.14)	0.920		0.97 (0.78–1.19)	0.747		1.05 (0.85–1.30)	0.631	
	rs4760663C > T	1.01 (0.89–1.14)	0.920		0.92 (0.75–1.13)	0.433		1.11 (0.90–1.38)	0.323	
*WNT3A*	rs13373831A > G	0.94 (0.80–1.10)	0.410		0.86 (0.71–1.04)	0.119		1.35 (0.87–2.08)	0.179	
	rs708113T > A	1.04 (0.91–1.18)	0.610		0.98 (0.81–1.18)	0.801		1.21 (0.93–1.58)	0.165	
	rs74672629C > G	0.92 (0.74–1.14)	0.430		0.90 (0.71–1.13)	0.352		1.28 (0.45–3.67)	0.644	
	**rs752107C>T**	**0**.**85** (**0**.**73**–**0**.**99**)	**0**.**040**	0.928	**0**.**82** (**0**.**68**–**0**.**98**)	**0**.**033**	0.766	0.86 (0.58–1.26)	0.433	
*WNT5A*	rs2076831C > G	1.03 (0.91–1.18)	0.610		1.07 (0.89–1.29)	0.461		1.00 (0.76–1.30)	0.93	
	rs3732750G > A	1.23 (1.00–1.52)	0.053		1.23 (0.98–1.55)	0.068		1.71 (0.63–4.60)	0.292	
	rs504849A > G	1.00 (0.87–1.14)	0.970		1.00 (0.83–1.20)	0.968		1.00 (0.77–1.30)	0.988	
	rs566926A > C	1.01 (0.89–1.16)	0.850		1.10 (0.91–1.32)	0.344		0.89 (0.68–1.15)	0.357	

**Table 4:** Haplotype association of SNPs in WNT pathway and tuberculosis.

Gene	Haplotype^a^	Frequency			OR (95% CI)	*P*	P**
		All	TB	HC			
*CTNNB1*	CGG	0.764	0.785	0.748	1.00		
	**GCA**	0.212	0.175	0.240	**0**.**69** (**0**.**60**–**0**.**83**)	**5**.**85 × 10**^**−4**^	**0**.**016**
*WNT5A*	AAC	0.617	0.614	0.619	1.00		
	CGG	0.359	0.366	0.354	1.03 (0.90–1.17)	0.700	
	CAC	0.014	0.019	0.010	2.04 (1.15–3.62)	0.054	
*WNT1*	CC	0.502	0.496	0.508	1.00		
	TT	0.475	0.480	0.47	1.01 (0.89–1.15)	0.840	
	CT	0.023	0.024	0.022	1.13 (0.73–1.76)	0.590	
*WIF1*	CC	0.819	0.830	0.811	1.00		
	GG	0.175	0.126	0.185	0.88 (0.74–1.03)	0.120	

P**: *p* value after Bonferroni correction.

^a^Haplotype frequency <0.01 in both cases and controls has been dropped; significant associations were denoted in bold. Wild haplotype was set as the reference, and the OR of other haplotypes were calculated.

**Table 5:** Association of six candidate SNPs with TB risk in Chinese Tibetan validation cohort.

SNP	Group	Genotype		Allele		Additive Model		Dominant Model		Recessive Model	
		(11/12/22)	P**	OR	P**	OR	P**	OR	P**	OR	P**
rs9859392	TB	19/131/327	**0**.**014**	**0**.**69** (**0**.**55**–**0**.**87**)	**0**.**016**	**0**.**70** (**0**.**56**–**0**.**88**)	**0**.**018**	**0**.**64** (**0**.**49**–**0**.**83**)	**0**.**010**	0.71 (0.39–1.32)	>0.999
	HC	24/159/254									
rs9870255	TB	19/130/328	**0**.**009**	**0**.**68** (**0**.**54**–**0**.**86**)	**0**.**011**	**0**.**70** (**0**.**55**–**0**.**87**)	**0**.**013**	**0**.**63** (**0**.**49**–**0**.**83**)	**0**.**008**	0.68 (0.37–1.26)	>0.999
	HC	25/158/254									
rs3864004	TB	19/130/328	**0**.**014**	**0**.**69** (**0**.**55**–**0**.**87**)	**0**.**015**	**0**.**70** (**0**.**56**–**0**.**88**)	**0**.**018**	**0**.**64** (**0**.**49**–**0**.**83**)	**0**.**010**	0.71 (0.39–1.32)	>0.999
	HC	24/158/255									
rs4736958	TB	33/206/238	>0.999	0.90 (0.74–1.10)	>0.999	0.90 (0.73–1.10)	>0.999	0.92 (0.71–1.19)	>0.999	0.74 (0.46–1.19)	>0.999
	HC	40/188/209									
rs7832767	TB	100/242/135	>0.999	0.93 (0.74–1.17)	>0.999	0.93 (0.73–1.18)	>0.999	0.90 (0.69–1.18)	>0.999	1.07 (0.49–2.34)	>0.999
	HC	91/233/113									
rs752107	TB	28/131/318	>0.999	1.18 (0.93–1.49)	>0.999	1.17 (0.93–1.47)	>0.999	1.06 (0.80–1.40)	>0.999	2.66 (1.27–5.55)	0.080
	HC	10/130/297									

Numbers were bolded if they showed a significance of *p* < 0.05; 11 = mutant homozygous, 12 = heterozygote, 22 = wild homozygous; OR = odds ratio, followed by 95% CI in parentheses. P**: *p* value after Bonferroni correction.
