# Supplementary material for: Pathway Analyses Identify Novel Variants in the WNT Signaling Pathway Associated with Tuberculosis in Chinese Population
Source: Sci Rep. 2016 Jun 23;6:28530. doi: 10.1038/srep28530 (PMC4917881; doi:10.1038/srep28530)
Supplement: Supplementary Information [file srep28530-s1.doc]

**Pathway Analyses Identify Novel Variants in the WNT Signaling Pathway Associated with Tuberculosis in Chinese Population**

Xuejiao Hu^1a^, Juan Zhou^1a^, Xuerong Chen^2a^, Yanhong Zhou^1^, Xingbo Song^1^, Bei Cai^1^, Jingya Zhang^1^, Xiaojun Lu^1^, Binwu Ying^1^*

^1^ Department of Laboratory Medicine, West China Hospital, Sichuan University, Chengdu 610041, P. R China

^2^ Division of Pulmonary Disease, West China Hospital, Sichuan University, Chengdu 610041, P. R China

**Running Title:** WNT Pathway polymorphisms associated with tuberculosis.

^a^. Xuejiao Hu, Juan Zhou and Xuerong Chen contributed equally to this article;

*. Corresponding author: Binwu Ying Ph.D

Department of Laboratory Medicine

West China Hospital, Sichuan University

Chengdu 610041, Sichuan Province, P.R China

Correspondence to [[docbwy@126.com](mailto:docybw@gmail.com)]

Tel: 86-28-85422751, Fax: 86-28-85422751

Supplementary Table 1. Characteristics of 25 SNPs used in WNT pathway analysis

| **Gene** | **SNP** | **Chr: position** | **Region** | **MAF in CHB** | **HWE-*P*** |
| --- | --- | --- | --- | --- | --- |
| *CTNNB1* | rs9859392 | 3: 41193025 | 5'-flanking | 0.256 | 0.751 |
|  | rs9870255 | 3: 41195090 | 5'-flanking | 0.256 | 0.750 |
|  | rs3864004 | 3: 41198686 | promoter | 0.275 | 0.524 |
|  | rs7630377 | 3: 41189163 | 5'-flanking | 0.302 | - |
| *WIF1* | rs34203757 | 12: 65121978 | promoter | 0.146 | >0.999 |
|  | rs34505206 | 12: 65122058 | promoter | 0.146 | >0.999 |
|  | rs56900803 | 12: 65122146 | promoter | 0.165 | 0.778 |
| *DKK1* | rs11001553 | 10: 52313141 | promoter | 0.128 | 0.129 |
|  | rs1896367 | 10: 52309426 | 5'-flanking | 0.372 | 0.204 |
|  | rs1896368 | 10: 52309144 | 5'-flanking | 0.465 | 0.231 |
| *SFRP1* | rs3242 | 8: 41262035 | 3'UTR | 0.056 | 0.153 |
|  | rs4736958 | 8: 41261978 | 3'UTR | 0.316 | 0.572 |
|  | rs72643819 | 8: 41309674 | promoter | 0.442 | 0.117 |
|  | rs72643820 | 8: 41309972 | promoter | 0.461 | - |
|  | rs7832767 | 8: 41302340 | intron | 0.256 | 0.750 |
| *WNT1* | rs4760662 | 12: 48977074 | promoter | 0.477 | 0.801 |
|  | rs4760663 | 12: 48974850 | promoter | 0.488 | 0.614 |
| *WNT3A* | rs13373831 | 1: 228006090 | promoter | 0.186 | 0.371 |
|  | rs708113 | 1: 228005052 | promoter | 0.267 | 0.489 |
|  | rs74672629 | 1: 228005692 | promoter | 0.083 | 0.550 |
|  | rs752107 | 1: 228059650 | 3'UTR | 0.221 | 0.622 |
| *WNT5A* | rs2076831 | 3: 55489010 | promoter | 0.354 | 0.894 |
|  | rs3732750 | 3: 55468223 | 3'UTR | 0.087 | 0.749 |
|  | rs504849 | 3: 55488911 | promoter | 0.364 | 0.791 |
|  | rs566926 | 3: 55486750 | promoter | 0.384 | 0.351 |

Note: Chr: chromosome;

MAF in CHB: minor allele frequency in Chinese Han Beijing population;

HWE-*P*: *P* value of Hardy-Weinberg equilibrium results in the present study.

**-** refers to failed genotyping, HWE-*P* could not be calculated.

Supplementary Table 2. Primers and probes of 25 SNPs for MassARRAY genotyping

| **Gene** | **SNP** | **Senseprimer** | **Antisenseprimer** | **Extensionprobe** |  |
| --- | --- | --- | --- | --- | --- |
| *CTNNB1* | rs9859392 | ACGTTGGATGCTTTTCCCAAATCGATTTTA | ACGTTGGATGGCTGACTGTGTCATTCTACC | CATTCTACCAAATGGAACTT |  |
|  | rs9870255 | ACGTTGGATGCTGTCTTTGGTTTGTTCATGC | ACGTTGGATGAGTGAAATTTCGCAGAGGGC | AAATCCCTGCTGTGCAGA |  |
|  | rs3864004 | ACGTTGGATGAGGACTTGTTGAATTGCGGG | ACGTTGGATGTTCTGTCCCCACTCACGAAG | CTGATGTGAACTCTCCGTAGAA |  |
|  | rs7630377 | ACGTTGGATGCACAAACCCATATCTGAGTG | ACGTTGGATGCACATTCTTACAGAATGTCC | GGTATAAACCACTCTATGTGCTCA |  |
| *WIF1* | rs34203757 | ACGTTGGATGTTCAGCCAGTAGGATTCCTC | ACGTTGGATGGTTTTAACTGCTTGGGAGCG | TGCCAGCCTATCGCA |  |
|  | rs34505206 | ACGTTGGATGAATGCTGGGTGTCGGGCAAG | ACGTTGGATGTAAAACTCGAGGCTCGACTG | CTGTTGCCGATGGCAGGCG |  |
|  | rs56900803 | ACGTTGGATGGAGGGTCAGGTACAGCTATC | ACGTTGGATGATTCCCGTCTGTCTCCCTGA | GACTTCTGTCTCCCTGATAACCC |  |
| *DKK1* | rs11001553 | ACGTTGGATGACCTTGAGGAGAAGAAGAGC | ACGTTGGATGTTCTTATACACCAGCCTTAC | ACCAGCCTTACTTTATTATATC |  |
|  | rs1896367 | ACGTTGGATGGTCTGTTCTTTTATGCAGCC | ACGTTGGATGACTTACATGCTAGTCTCTTC | TGCGAGTCTCTTCAAAATCGACATG |  |
|  | rs1896368 | ACGTTGGATGGTCTTGACACCCAGTCTTAG | ACGTTGGATGATACATCCCTCCTGCTCTTC | CCTACATCTTCCACTCTT |  |
| *SFRP1* | rs3242 | ACGTTGGATGCATGGCTGAAAAAGCATGAA | ACGTTGGATGCCAGATGTTTTGATGTTATCG | TTATGTTAATAGTAATTCCCGTA |  |
|  | rs4736958 | ACGTTGGATGTGCTTTTTCAGCCATGTATC | ACGTTGGATGAAGTCACAGCTCACAGTATC | CACAGTATCATTGATTAATTGAGTGA |  |
|  | rs72643819 | ACGTTGGATGCGGCTCAACACCCCTTAAAA | ACGTTGGATGCCCTTCTTTTTCTCCCCTTG | TTTCGCCCCTTGTCTCTTTCCT |  |
|  | rs72643820 | ACGTTGGATGTAACATGGTGAAACCCCGTC | ACGTTGGATGAGTAGCTGGGATTACAGCCG | ACAGCCGCGTGCCACCA |  |
|  | rs7832767 | ACGTTGGATGCAATACCCTGAGAATGACCC | ACGTTGGATGAGTAAGCTGCACACATGTGG | ACCATGCAAGTCGGAGG |  |
| *WNT1* | rs4760662 | ACGTTGGATGATGGATGAGATGGTCACAGG | ACGTTGGATGGCCAAGTTTCTGTCTTTCCC | CCACCACACTGGGATA |  |
|  | rs4760663 | ACGTTGGATGGAGCATGCTTGGTTCAAAGG | ACGTTGGATGCTTCATTGCCTACCACTCTC | CACTACCACTCTCTTCCTTG |  |
| *WNT3A* | rs13373831 | ACGTTGGATGACTTTCCCTCTCAGGTCTTC | ACGTTGGATGTGTTCCGACAGAGGGATTTC | TGTAGTTAAGCTTTAGGAGTTTGC |  |
|  | rs708113 | ACGTTGGATGTTAGAGCCCCCTAGAGGATG | ACGTTGGATGACTGAGCAGGCTGCGCTCAC | GTTAACCTGCCCCCTTCAG |  |
|  | rs74672629 | ACGTTGGATGATCCCTGCCTCTCCATGCAC | ACGTTGGATGTGTGTTCTCTGGGGATACTG | AGGGACGGCTGCAGAGTTGC |  |
|  | rs752107 | ACGTTGGATGGGCAGAACTCCTACCTGAAG | ACGTTGGATGTCATTCAGGAGCAGCCCAG | CCAGAGAGGAGACACT |  |
| *WNT5A* | rs2076831 | ACGTTGGATGAACTTCGCAATCTCCTCACC | ACGTTGGATGATGATTGCTCATCTGGCTCC | GATTGCTCCGGCCCAGAGCAT |  |
|  | rs3732750 | ACGTTGGATGGCTATCTATGTAGTGGGCTG | ACGTTGGATGTAGTTCATTCTGCAGAATGG | GGGATGCAGAATGGAAACCCATG |  |
|  | rs504849 | ACGTTGGATGTACCCAAGTCCCTACTACTC | ACGTTGGATGGGAGGATACTTAAGCACTGG | AAGCACTGGGGCTGA |  |
|  | rs566926 | ACGTTGGATGGTGTCCCAGGAGATCACTTC | ACGTTGGATGTCGCTCTTCCCCACTTTTTC | TCCACACTTTTTCTCAAAAATCAAAAT |  |

**Supplementary Table 3. Primers of susceptible SNPs for the multiplex PCR reaction**

| **SNPs** | **Primer** | **Sequences (5'→3')** |
| --- | --- | --- |
| rs3864004 | F | GCGCTCTGGAGCTAATCCATTTC |
|  | R | CCCACTCACGAAGGCTGTGAAC |
| rs9859392 | F | TGGACAATCCCTTGAAGCAACA |
|  | R | TGTAGAATTTGAGTTGGGGGAAAAGA |
| rs9870255 | F | TGCAGTGCTAGGGTGGTGAGTG |
|  | R | AGGGTCCACGGTTCCCATTTAG |
| rs4736958 | F | GTGGTGCCTCCCAAGTTCTCCT |
|  | R | TTCATGCTTTTTCAGCCATGTATCA |
| rs7832767 | F | CCTGAGAATGACCCTGTCGTGAG |
|  | R | GGCATCCCAGTCCATGTTTTCA |
| rs752107 | F | GGGGGCAGAACTCCTACCTGAA |
|  | R | AATCTGTAGCCCCGCCTCTGTC |

**Supplementary Table 4. The probe sequences for multiplex ligation detection reaction method**

| **Probe Name** | | **Target Allele** | **Probe Sequence (5' phosphorated)** | |
| --- | --- | --- | --- | --- |
| rs3864004FG | G | | | pTCTCTCGGGTCAATTCGTCCTTAATTGCGGGCTTGGCGCACG |
| rs3864004FA | A | | | pTGTTCGTGGGCCGGATTAGT AATTGCGGGCTTGGCGCACA |
| rs3864004FP |  | | | TTCTACGGAGAGTTCACAGCCTTCTTTTTTTTT |
| rs9859392RC | C | | | pTTCCGCGTTCGGACTGATATTGACTGTGTCATTCTACCAAATGGAACATG |
| rs9859392RG | G | | | pTACGGTTATTCGGGCTCCTGTTGACTGTGTCATTCTACCAAATGGAACATC |
| rs9859392RP |  | | | CTGAAATCATATAGTTCTTTAATTATAAAATCGATTTG |
| rs9870255FG | G | | | pTTCCGCGTTCGGACTGATATTGTCTTTGGTTTGTTCATGCTTTTGAG |
| rs9870255FC | C | | | pTACGGTTATTCGGGCTCCTGTTGTCTTTGGTTTGTTCATGCTTTTGAC |
| rs9870255FP |  | | | TCTGCACAGCAGGGATGTTCTTTTTTTTT |
| rs4736958FC | C | | | pTCTCTCGGGTCAATTCGTCCTTCACAGCTCACAGTATCATTGATTAATTGAGTCAC |
| rs4736958FT | T | | | pTGTTCGTGGGCCGGATTAGTCACAGCTCACAGTATCATTGATTAATTGAGTCAT |
| rs4736958FP |  | | | TTTAGTCAAGTGAATATTGATACATGGCTGTTTT |
| rs7832767FC | C | | | pTCTCTCGGGTCAATTCGTCCTTGACCCTGTCGTGAGGCATGAGTC |
| rs7832767FT | T | | | pTGTTCGTGGGCCGGATTAGTGACCCTGTCGTGAGGCATGAGTT |
| rs7832767FP |  | | | CCTCYGACTTGCATGTGTCCTTTT |
| rs752107FC | C | | | pTTCCGCGTTCGGACTGATATCAGGGCTCCTCCCTGGATCC |
| rs752107FT | T | | | pTACGGTTATTCGGGCTCCTGTCAGGGCTCCTCCCTGGACCT |
| rs752107FP |  | | | AGTGTCTCCTCTCTGGTGGCTGTTTTT |
|  | |  | |  |

**Supplementary Table 5. Primers of candidate genes for mRNA measurement**

| **Gene** | **Primer** | **Sequences (5'→3')** |
| --- | --- | --- |
| ***SFRP1*** | F | CGAGTTTGCACTGAGGATGA |
|  | R | CAGCACAAGCTTCTTCAGGTC |
| ***WNT3A*** | F | TGGTGTCTCGGGAGTTCGC |
|  | R | CCGTGGCACTTGCACTTGA |
| ***CTNNB1*** | F | GCTGATTTGATGGAGTTGGA |
|  | R | GCTACTTGTTCTTGAGTGAA |
| ***GAPDH*** | F | TCACCAACTGGGACGACATG |
|  | R | ACCGGAGTCCATCACGATG |

**Supplementary Table 6. Association of candidate SNPs with tubercular subtypes in Chinese Han population**

| **SNP** | **Group** | **Genotype** | |  | **Allele** | |  | **Additive Model** | |  | **Dominant Model** | |  | **Recessive Model** | |
| --- | --- | --- | --- | --- | --- | --- | --- | --- | --- | --- | --- | --- | --- | --- | --- |
|  |  | (11/12/22) | *P*** |  | OR | *P*** |  | OR | *P*** |  | OR | *P*** |  | OR | *P*** |
| rs4736958 | Control | 125/477/478 |  |  |  |  |  |  |  |  |  |  |  |  |  |
|  | All TB | 79/313/426 | 0.092 |  | **0.78 (0.69-0.92)** | **0.033** |  | **0.78 (0.70-0.92)** | **0.040** |  | **0.73 (0.61-0.88)** | **0.017** |  | 0.82 (0.61-1.10) | >0.999 |
|  | PTB | 27/130/200 | **0.008** |  | **0.68 (0.60-0.83)** | **0.001** |  | **0.69 (0.57-0.83)** | **0.001** |  | **0.62 (0.49-0.79)** | **0.001** |  | 0.63 (0.41-0.97) | 0.204 |
| rs9859392 | Control | 60/395/607 |  |  |  |  |  |  |  |  |  |  |  |  |  |
|  | All TB | 38/240/536 | **0.013** |  | **0.75 (0.64-0.88)** | **0.009** |  | **0.76 (0.65-0.88)** | **0.010** |  | **0.69 (0.57-0.84)** | **0.003** |  | 0.82 (0.54-1.24) | >0.999 |
|  | PTB&EPTB | 13/100/228 | **0.011** |  | **0.71 (0.57-0.88)** | **0.010** |  | **0.71 (0.57-0.88)** | **0.011** |  | **0.66 (0.51-0.85)** | **0.009** |  | 0.66 (0.36-1.22) | 0.186 |
| rs9870255 | Control | 64/394/609 |  |  |  |  |  |  |  |  |  |  |  |  |  |
|  | All TB | 34/255/525 | 0.092 |  | **0.76 (0.65-0.89)** | **0.018** |  | **0.77 (0.65-0.90)** | **0.019** |  | **0.73 (0.61-0.88)** | **0.026** |  | 0.68 (0.45-1.05) | >0.999 |
|  | PTB&EPTB | 14/99/230 | 0.087 |  | **0.70 (0.57-0.87)** | **0.007** |  | **0.70 (0.57-0.88)** | **0.009** |  | **0.65 (0.51-0.84)** | **0.007** |  | 0.67 (0.37-1.21) | >0.999 |
| rs3864004 | Control | 67/375/610 |  |  |  |  |  |  |  |  |  |  |  |  |  |
|  | All TB | 36/250/527 | 0.149 |  | **0.77 (0.66-0.91)** | **0.033** |  | **0.78 (0.67-0.91)** | **0.038** |  | 0.75 (0.62-0.90) | 0.063 |  | 0.68 (0.45-1.03) | >0.999 |
|  | PTB&EPTB | 14/98/228 | 0.211 |  | **0.71 (0.57-0.89)** | **0.014** |  | **0.72 (0.58-0.89)** | **0.017** |  | **0.68 (0.52-0.88)** | **0.018** |  | 0.63 (0.35-1.14) | >0.999 |
| rs7832767 | Control | 68/393/617 |  |  |  |  |  |  |  |  |  |  |  |  |  |
|  | All TB | 89/305/426 | **0.021** |  | **1.30 (1.11-1.48)** | **0.016** |  | **1.29 (1.10-1.46)** | **0.023** |  | 1.26 (1.03-1.49) | 0.505 |  | **1.81 (1.30-2.52)** | **0.010** |
|  | PTB&EPTB | 44/138/161 | **0.001** |  | **1.51 (1.25-1.82)** | **0.001** |  | **1.49 (1.23-1.79)** | **0.001** |  | 1.53 (1.19-1.93) | 0.063 |  | **2.19 (1.47-3.26)** | **0.001** |

Note: Numbers were bolded if they showed a significance of *p* <0.05; 11 = mutant homozygous, 12 = heterozygote, 22 = wild homozygous; OR = odds ratio, followed by 95% CI in parentheses; P**: *p* value after Bonferroni correction.

Supplementary Table 7. Multiplicative gene-gene interactions for different gene polymorphisms

| **Genotype** | **Genotype** | **Case/Control (n/n)** | **OR (95% CI)** | ***P*_boot_** |
| --- | --- | --- | --- | --- |
| *rs3864004 and rs4736958* | | | |  |
| AG+AA | TC+CC | 190/346 | 1 |  |
| AG+AA | TT | 88/79 | 1.51 (1.16-1.96) |  |
| GG | TC+CC | 186/225 | 1.63 (1.15-2.32) |  |
| GG | TT | 373/329 | 1.61( 1.27-2.02) | 0.653 |
| *rs3864004 and rs752107* | | | |  |
| AG+AA | TC+TT | 182/333 | 1 |  |
| AG+AA | CC | 87/101 | 1.50 (1.19-1.88) |  |
| GG | TC+TT | 161/158 | 1.58 (1.12-2.21) |  |
| GG | CC | 357/437 | 1.79 (1.35-2.39) | 0.103 |
| *rs3864004 and rs7832767* | | | |  |
| AG+AA | TC+CC | 220/403 | 1 |  |
| AG+AA | TT | 49/31 | 1.51 (1.23-1.85) |  |
| GG | TC+CC | 477/578 | 2.89 (1.79-4.67) |  |
| GG | TT | 38/20 | 3.48 (1.98-6.13) | 0.154 |
| *rs4736958 and rs752107* | | | |  |
| TC+TT | TC+TT | 248/398 | 1 |  |
| TC+TT | CC | 128/173 | 1.38 (1.12-1.73) |  |
| TT | TC+TT | 92/96 | 1.18 (0.89-1.56) |  |
| TT | CC | 316/365 | 1.54 (1.11-2.13) | 0.209 |
| *rs4736958 and rs9859392* | | | |  |
| TC+CC | GC+GG | 178/348 | 1 |  |
| TC+CC | CC | 198/223 | 1.79 (1.26-2.56) |  |
| TT | GC+GG | 80/87 | 1.73(1.33-2.25) |  |
| TT | CC | 328/374 | 2.02(1.48-3.05) | 0.075 |
| rs4736958 and rs9870255 | | | |  |
| TC+CC | GC+CC | 193/349 | 1 |  |
| TC+CC | GG | 183/222 | 1.79 (1.26-2.56) |  |
| TT | GC+CC | 79/88 | 1.73(1.33-2.25) |  |
| TT | GG | 329/373 | 2.02(1.48-3.05) | 0.075 |
| *rs752107and rs7832767* | | | |  |
| TC+TT | CC | 287/456 | 1 |  |
| TC+TT | TC+TT | 53/38 | 1.32 (0.95-2.12) |  |
| CC | CC | 410/525 | 1.59 (1.08-2.34) |  |
| CC | TC+TT | 34/13 | 1.62 (1.14-2.31) | 0.086 |
| *rs752107and rs9859392* | | | |  |
| TC+TT | GC+GG | 169/336 | 1 |  |
| TC+TT | CC | 171/158 | 1.61 (1.28-2.03) |  |
| CC | GC+GG | 89/99 | 1.79 (1.27-2.51) |  |
| CC | CC | 355/439 | 2.15 (1.62-2.86) | 0.121 |
| rs752107and rs9870255 | | | |  |
| TC+TT | GC+CC | 185/336 | 1 |  |
| TC+TT | GG | 155/158 | 1.56 (112-2.19) |  |
| CC | GC+CC | 87/101 | 1.48 (1.18-1.86) |  |
| CC | GG | 357/437 | 1.78 (1.34-2.37) | 0.180 |
| *rs7832767 and rs9859392* | | | |  |
| CC | GC+GG | 210/403 | 1 |  |
| CC | CC | 487/578 | 1.76 (1.15-2.53) |  |
| TC+TT | GC+GG | 48/32 | 1.23 (0.75-2.10) |  |
| TC+TT | CC | 39/19 | 1.78 (1.04-2.59) | 0.657 |
| *rs7832767 and rs9870255* | | | |  |
| CC | GC+CC | 405/226 | 1 |  |
| CC | GG | 471/576 | 2.57 (1.59-4.16) |  |
| TC+TT | GC+CC | 46/32 | 1.46 (1.20-1.84) |  |
| TC+TT | GG | 41/19 | 3.86 (2.20-6.82) | 0.073 |

Supplementary Table 8. Additive gene-gene interactions for different gene polymorphisms

| **Measure** | **Estimate** | **Lower** | **Upper** |  | **Measure** | **Estimate** | **Lower** | **Upper** |  | **Measure** | **Estimate** | **Lower** | **Upper** |
| --- | --- | --- | --- | --- | --- | --- | --- | --- | --- | --- | --- | --- | --- |
| *rs3864004 and rs4736958* | | | |  | *rs4736958 and rs9859392* | | | |  | *rs7832767 and rs9859392* | | | |
| RERI | -0.534 | -1.217 | 0.148 |  | RERI | 0.194 | -0.831 | 0.325 |  | RERI | -0.041 | -0.657 | 0.579 |
| AP | -0.333 | -0.760 | 0.094 |  | AP | 0.363 | -0.061 | 0.762 |  | AP | -0.052 | -0.498 | 0.497 |
| S | 0.532 | 0.278 | 1.015 |  | S | 1.932 | 1.184 | 2.810 |  | S | 0.934 | 0.221 | 2.166 |
| *rs3864004 and rs752107* | | | |  | *rs752107 and rs7832767* | | | |  | Note: Measurements of additive gene interactions-relative excess risk due to interaction (RERI); attributable proportion due to interaction (AP); and synergy index (SI). The three indices designate significant interactions when they differ from 0 for RERI and AP, or 1 for SI. | | | |
| RERI | -0.876 | -1.605 | -0.048 |  | RERI | -0.433 | -1.19 | 0.289 |  |
| AP | -0.586 | -1.074 | -0.099 |  | AP | -0.472 | -0.763 | 0.245 |  |
| S | 0.361 | 0.189 | 0.689 |  | S | 0.592 | 0.237 | 1.615 |  |
| *rs3864004 and rs7832767* | | | |  | *rs752107 and rs9870255* | | | |  |
| RERI | 0.073 | -2.203 | 2.350 |  | RERI | 0.054 | -0.573 | 0.637 |  |
| AP | 0.021 | -0.624 | 0.666 |  | AP | 0.028 | -0.119 | 0.296 |  |  |  |  |  |
| S | 1.031 | 0.407 | 2.606 |  | S | 1.038 | 0.585 | 1.973 |  |  |  |  |  |
| *rs4736958 and rs752107* | | | |  | *rs752107 and rs9859392* | | | |  |  |  |  |  |
| RERI | -0.335 | -0.924 | 0.253 |  | RERI | -0.083 | -0.553 | 0.926 |  |  |  |  |  |
| AP | -0.241 | -0.664 | 0.181 |  | AP | -0.048 | -0.471 | 0.376 |  |  |  |  |  |
| S | 0.537 | 0.227 | 1.272 |  | S | 1.134 | 0.522 | 2.07 |  |  |  |  |  |
| *rs4736958 and rs9870255* | | | |  | *rs7832767 and rs987255* | | | |  |  |  |  |  |
| RERI | 0.421 | -0.478 | 1.356 |  | RERI | -0.196 | -0.929 | 0.456 |  |  |  |  |  |
| AP | 0.327 | -0.281 | 0.658 |  | AP | -0.186 | -0.721 | 0.435 |  |  |  |  |  |
| S | 1.869 | 0.434 | 7.969 |  | S | 0.813 | 0.347 | 1.970 |  |  |  |  |  |

**Supplementary Table 9. High-dimensional interactions among all SNPs by GMDR ^[1]^**

| **Model** | **SNPs** | **Training. BA** | **Testing. BA** | ***P*** | **CV consistency** |
| --- | --- | --- | --- | --- | --- |
| one-way | rs9839392 | 0.584 | 0.563 | 0.055 | 7/10 |
| two-way | rs3864004 rs9859392 | 0.569 | 0.537 | 0.055 | 9/10 |
| three-way | rs3864004 rs74672629 rs9859392 | 0.583 | 0.569 | 0.055 | 8/10 |
| four-way | rs3864004 rs4736958 rs74672629 rs9859392 | 0.571 | 0.541 | 0.217 | 10/10 |

Note: Training. BA refers to training balanced accuracy, Testing. BA indicates testing balanced accuracy.

[1] Tunesi, S., et al. Gene-asbestos interaction in malignant pleural mesothelioma susceptibility. *Carcinogenesis* **36**, 1129-1135 (2015).
